# Aortic dissection in a patient treated with anlotinib for metastatic lung squamous cell carcinoma

**DOI:** 10.1111/1759-7714.13288

**Published:** 2019-12-31

**Authors:** Bailing Jiang, Junhe Li, Jun Chen, Xiaojun Xiang, Jianping Xiong, Jun Deng

**Affiliations:** ^1^ Department of Oncology First Affiliated Hospital of Nanchang University Nanchang China

**Keywords:** Anlotinib, aortic dissection, lung squamous cell carcinoma

## Abstract

Anlotinib is an anti‐angiogenic drug that targets vascular endothelial growth factor receptor, platelet‐derived growth factor receptor, fibroblast growth factor receptor, c‐Kit, and other kinases and has been approved for the treatment of advanced non‐small cell lung cancer (NSCLC). As in other small‐molecule tyrosine kinase inhibitors, adverse effects such as hypertension and cardiotoxicity may be seen. However, the relationship between anlotinib and aortic dissection has not been previously reported. Here, we present a case of aortic dissection in a 58‐year‐old male patient with advanced NSCLC without history of hypertension who received anlotinib as third‐line treatment. After four courses of anlotinib treatment, he suffered a sudden onset of back pain, sweating, anxiety, and high blood pressure (180/120 mmHg) and heart rate (137 bpm). Emergency computed tomographic angiography revealed aortic dissection and thrombosis of the distal false lumen. Thereafter, the patient was administered nitroglycerin as antihypertensive treatment and he underwent stent‐graft intervention for aortic dissection. Anticoagulants and antihypertensive drugs were administered after the operation, and a moderate control of blood pressure was achieved. Thus, the adverse reactions of antolinib must be monitored and clinicians must be vigilant.

## Introduction

Lung cancer is the most common and aggressive solid tumor worldwide which accounts for 11.6% of the whole cancer population and contributes to 18.4% of cancer‐related death.^1^ Anlotinib hydrochloride (AL3818) is an innovative small‐molecule multi‐target tyrosine kinase inhibitor which can effectively inhibit vascular endothelial growth factor receptor (VEGFR), platelet‐derived growth factor receptor (PDGFR), fibroblast growth factor receptor 1‐4 (FGFR 1‐4), c‐Kit, and other kinases, thereby exerting antitumor angiogenesis and tumor growth inhibition effects.^2^ The ALTER0303 study confirmed that as a third‐line treatment for advanced non‐small cell lung cancer (NSCLC), anlotinib could achieve dual benefits for overall survival (9.63 months vs. 6.30 months, *P* < 0.01) and progression‐free survival (5.37 months vs. 1.40 months, *P* < 0.0001) compared to the placebo group, and its safety was controllable.^3^ The common adverse reactions of anlotinib include hypertension, fatigue, elevated thyroid‐stimulating hormone, hypercholesterolemia, hand‐foot syndrome, and hypertriglyceridemia.^4^ In this article, we report a case of aortic dissection potentially caused by anlotinib in stage IV lung squamous cell carcinoma and focus on the cardiotoxicity of anlotinib.

## Case report

**Figure 1:**
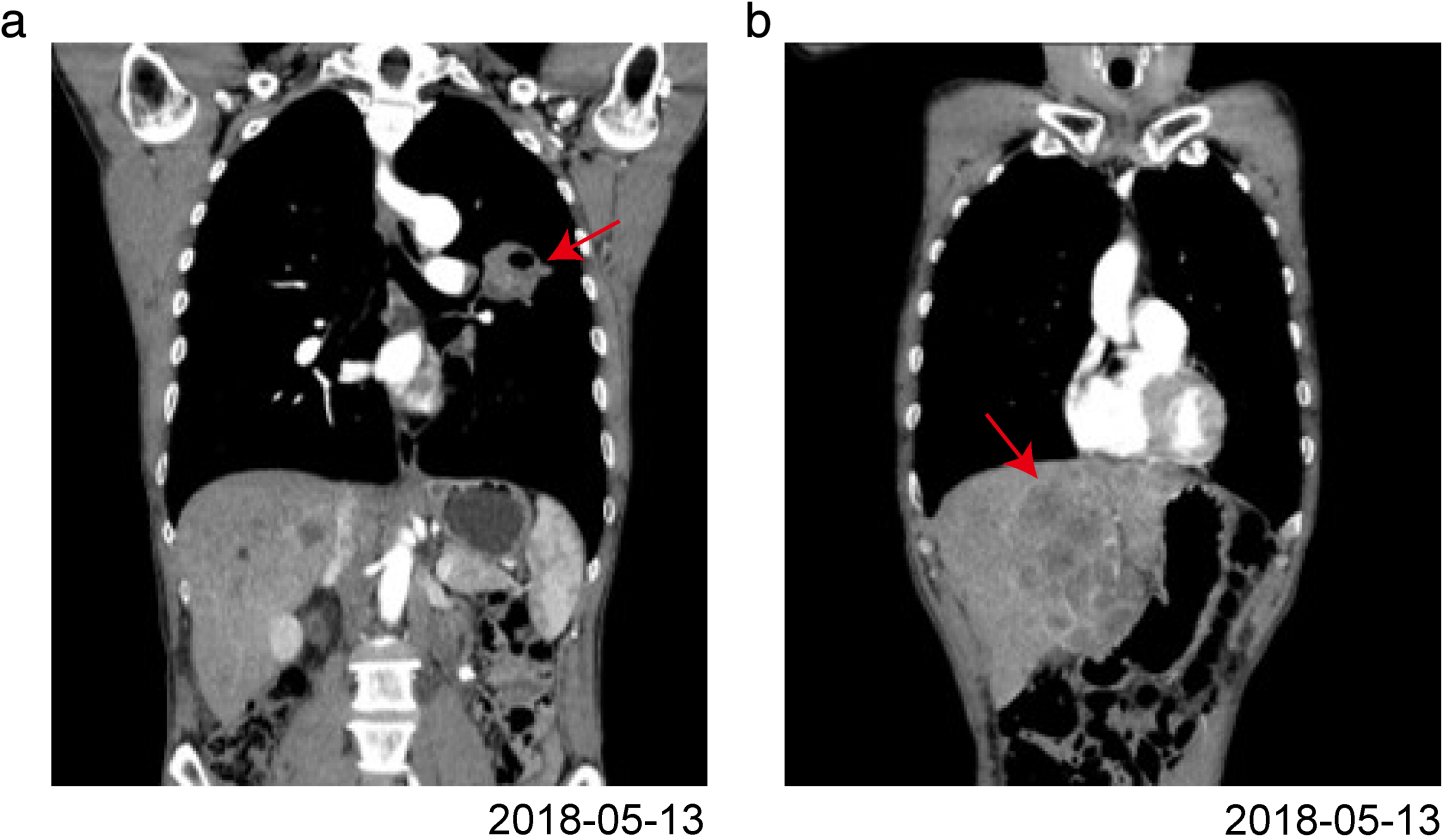
Computed tomography before treatment (**a**) primary lesion located in the upper left lung with left hilum lymph node metastasis, (**b**) multiple metastasis seen in the liver.

A 58‐year‐old male patient presented with cough producing sputum mixed with blood in May 2018 at the First Affiliated Hospital of Nanchang University. The patient had no history of hypertension or heart disease but had a history of nephrolithiasis without other abnormalities. Computed tomography revealed primary lung cancer in the upper left lung with left hilar lymph node metastasis and multiple metastatic tumors in the liver (Fig 1). Pathological examination of the bronchial biopsy revealed lung squamous cell carcinoma. Cranial magnetic resonance imaging (MRI) or bone scan revealed no significant abnormality. The patient was subsequently diagnosed with stage IV lung squamous cell carcinoma. Next‐generation sequencing (NGS) of lung cancer tissues did not show EGFR, ALK, ROS1, MET, BRAF V600E or NTRK gene mutation. Immunohistochemistry of lung cancer tissues showed PD‐L1 tumor proportion score of 5%.

In May 2018, the patient received first‐line combined chemotherapy with four courses of gemcitabine and cisplatin. Thereafter, progressive disease (PD) was found in the lung and liver lesions. In August 2018, docetaxel monotherapy was administered and he developed PD after two courses of chemotherapy. In October 2018, third‐line targeted therapy with anlotinib (12 mg once a day orally for two weeks, every three weeks) for four courses was commenced. During the treatment period, re‐examination showed that the tumors were stable. The adverse reactions were Grade 2 fatigue, Grade 1 oral mucositis, Grade 2 hand‐foot syndrome, and Grade 2 hypertriglyceridemia (according to CTCAE 5.0 criteria), which could resolve after drug withdrawal. During this period, blood pressure was normal, with an occasional increase to 160/72 mm of Hg (Grade 3); no further significant abnormalities were noted on electrocardiogram.

**Figure 2:**
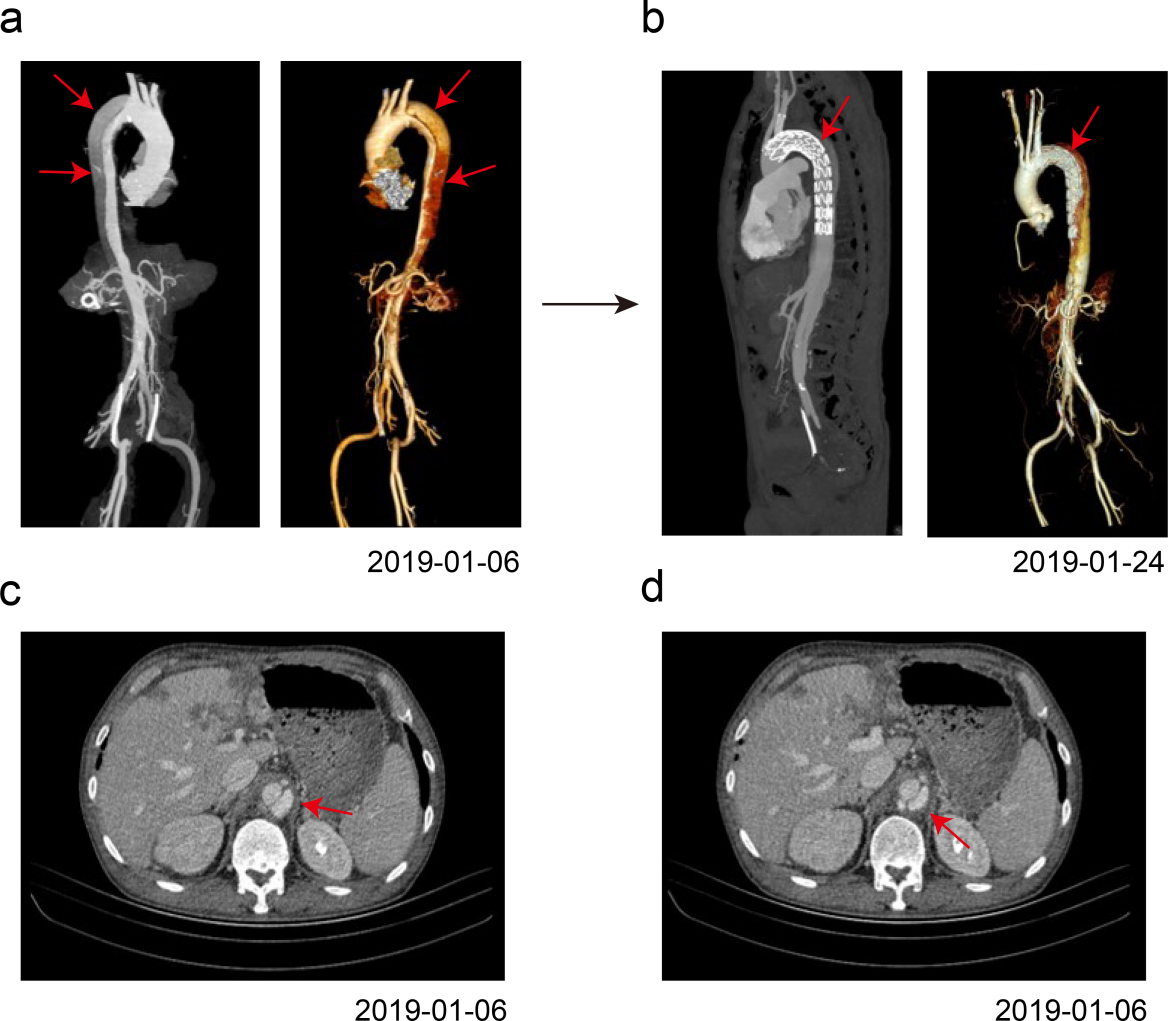
Computed tomographic angiography scan after anlotinib therapy (**a**) aortic dissection (DeBakey type IIIb), (**b**) stent‐graft implanted into the aorta; (**c**, **d**): thrombosis of the distal false lumen is observed.

**Figure 3:**
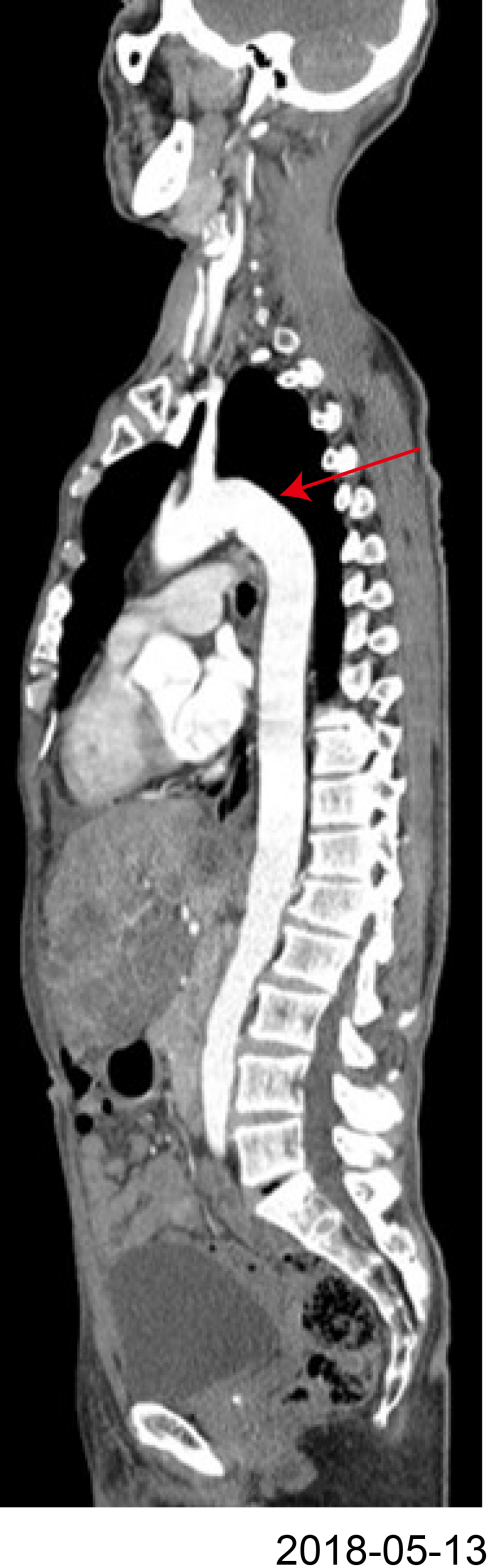
Computed tomography of thoracic aorta without aortic dissection before treatment.

In January 2019, the patient experienced a sudden onset of back pain, sweating, and anxiety; his blood pressure increased to 180/120 mmHg and his heart rate was 137 bpm. Emergency computed tomographic angiography revealed aortic dissection (DeBakey type IIIb) (Fig 2a) and thrombosis of the distal false lumen (Fig 2c,d). There were no signs of aortic dissection, thoracic aortic aneurysm, or dilatation of the aortic root before treatment on CT scan images (Fig 3). Thereafter, the patient received nitroglycerin as antihypertensive medication, and stent‐graft intervention was performed for aortic dissection (Fig 2b). Anticoagulants and antihypertensive drugs were applied after the operation, and blood pressure control was fair. In February 2019, the patient received nivolumab as fourth‐line treatment. Unfortunately, the patient developed hyperprogression disease (HPD) after two courses of treatment according to immune response evaluation criteria in solid tumor (iRECIS). After that, he died of respiratory failure.

## Discussion

This is the first report on aortic dissection potentially related to the treatment of anlotinib in advanced lung cancer. Previous studies have also shown that anti‐angiogenic monoclonal antibodies and small‐molecule TKI can lead to aortic dissection^5^, ^6^, ^7^, ^8^, ^9^, ^10^ (Table 1).

**Table 1:** Cases of aortic dissection during TKI or anti‐angiogenic monoclonal antibodies and small‐molecule TKI therapy

Drug	Tumor type	Authors	Reference number
Sunitinib	Renal cell carcinoma	Edeline *et al*.	17
bevacizumab, docetaxel, thalidomide, and prednisone	prostate cancer	Aragon‐Ching *et al*.	18
Sunitinib	Gastrointestinal stromal tumor	Hatem *et al*.	5
Axitinib	Renal cell carcinoma	Niwa *et al*.	6
Sorafenib and axitinib	Renal cell carcinoma	Takada *et al*.	7
Pazopanib, lapatinib and sunitinib	Renal cell carcinoma	Funahashi *et al*.	8
Sunitinib	Renal carcinoma	Formiga *et al*.	9
Sorafenib	Hepatocellular carcinoma	Xu *et al*.	10

The main causes of aortic dissection include older age, dyslipidemia, hypertension, atherosclerosis, previous cardiac surgery, iatrogenic causes, connective tissue disorders, thoracic aortic aneurysm, and dilatation of the aortic root.^11^ Hypertension and dyslipidemia are the main inducing factors of aortic dissection and main adverse reactions of anlotinib^4^ with complex correlations among these factors.

The molecular mechanism underlying the pathogenesis of aortic dissection caused by anlotinib is still unclear. The aorta is comprised of the tunica intima, tunica media, and tunica adventitia.^12^ Vascular endothelial growth factor receptor (VEGFR), platelet‐derived growth factor receptor (PDGFR) and fibroblast growth factor receptor (FGFR1‐4) are expressed in vascular endothelial cells and maintain normal vascular endothelial development and homeostasis.^13^, ^14^, ^15^, ^16^ Anlotinib is an anti‐angiogenic drug that targets VEGFR, PDGFR, FGFR1‐4, c‐Kit, and other kinases and has been approved for the treatment of advanced non‐small cell lung cancer (NSCLC).^2^, ^3^ In this case, the patient has no history of hypertension with only occasional hypertension during treatment. Based on the fact that he had Grade 2 hyperlipidemia caused by anlotinib, we speculated that both the anlotinib‐related hyperlipidemia and inhibition of VEGFR, PDGFR and FGFR by anlotinib may have contributed to aortic dissection.

As a new drug, its adverse reactions should be closely monitored, in particular for cardiotoxicity‐aortic dissection, and currently there is no a good monitoring indicator. Clinicians should closely monitor blood pressure, dyslipidemia and clinical symptoms such as chest pain. Appropriate management of TKI‐induced toxicities are essential to prevent cardiovascular events.

## Disclosure

The authors declare no conflict of interest.
